# Salisylic Acid as an Anti-Scorbutic

**Published:** 1879-12

**Authors:** 


Salisylic Acid as an Anti-Scorbutic.—Dr. L. G. Lincecum, of Texas, would call the attention of the profession to the use of salicylic acid as an anti-scorbutic. I have used it in seven cases with perfect success. Given internally one gramme, daily, in solution; with carbolated water as an external application. I hope the profession will give it a trial in all scorbutic troubles.

